# RGD-HK Peptide-Functionalized Gold Nanorods Emerge as Targeted Biocompatible Nanocarriers for Biomedical Applications

**DOI:** 10.1186/s11671-018-2828-3

**Published:** 2019-01-08

**Authors:** Sohameh Mohebbi, Tahereh Tohidi Moghadam, Maryam Nikkhah, Mehrdad Behmanesh

**Affiliations:** 1 Department of Nanobiotechnology, Faculty of Biological Sciences, Tarbiat Modares University, P.O. Box: 14115-154, Tehran, Iran; 2 Department of Genetics and Nanobiotechnology, Faculty of Biological Sciences, Tarbiat Modares University, P.O. Box: 14115-154, Tehran, Iran

**Keywords:** Gold nanorods, Peptide functionalization, Biocompatibility, Drug delivery systems, Targeting

## Abstract

**Abstract:**

Gold nanorods (GNRs) have been nominated as a promising candidate for a variety of biological applications; however, the cationic surfactant layer that surrounds a nanostructure places limits on its biological applicability. Herein, CTAB-GNRs were functionalized via a ligand exchange method using a (C(HK)4-mini PEG-RGD)-peptide to target the overexpressed αvβ3 integrin in cancerous cells, increase the biocompatibility, and gain the ability of gene/drug delivery, simultaneously. To confirm an acceptable functionalization, UV–Visible, FTIR, and Raman spectroscopy, zeta potential, and transmission electron microscopy of nanostructures were done. MTT assay was applied to study the cytotoxicity of nanostructures on two cell lines, HeLa and MDA-MB-231, as positive and negative αvβ3 integrin receptors, respectively. The cytotoxic effect of peptide-functionalized GNRs (peptide-f-GNRs) was less than that of CTAB-coated GNRs (CTAB-GNRs) for both cell lines. Uptake of peptide-f-GNRs and CTAB-GNRs was evaluated in two cell lines, using dark-field imaging and atomic absorption spectroscopy. Peptide-f-GNRs showed a proper cell uptake on the HeLa rather than MDA-MB-231 cell line according to the RGD (Arg-Gly-Asp) sequence in the peptide. The ability of peptide-f-GNRs to conjugate to antisense oligonucleotides (ASO) was also confirmed using zeta potential, which was due to the repeated HK (His-Lys) sequence inside the peptide. The result of these tests highlights the functionalization method as a convenient and cost-effective strategy for promising applications of targeted GNRs in the biological gene/drug delivery systems, and the repeated histidine-lysine pattern could be a useful carrier for negatively charged drug/gene delivery, too.

**Graphical Abstract:**

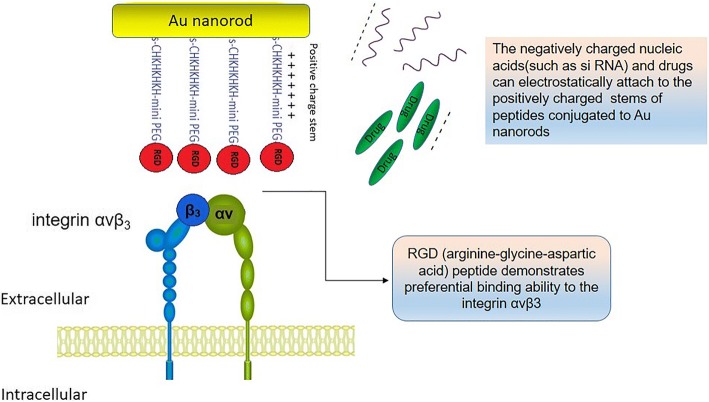

## Introduction

A lack of targeted imaging and therapy is an important issue in cancer treatment. This means that the harmful side effects and off-target of drugs can damage healthy cells during treatment. To resolve this problem, various nanocarriers such as liposomes, micelles, and metal nanoparticles have been used to reach specific cells or organs of interest using a targeting ligand such as cell penetrating peptides, folate, or a short RGD peptide sequence grafted to the surface [1]. RGD (arginine-glycine-aspartic acid) peptide demonstrates preferential binding ability to the integrin αvβ_3_. The role of integrins in the migration and invasion of tumors has been demonstrated, for a long time [2]. RGD-targeted nanocarriers present a promising approach for targeting tumor cells or tumor vasculature to deliver anticancer drugs or contrast agents for theranostics. Such nanocarriers could enter specific target cells via receptor-mediated endocytosis [3, 4]. The extracellular matrix (ECM) proteins, growth factors, cytokines, immunoglobulin, and matrix-degrading proteases have RGD sequence that interacts with integrin receptors at the focal region of adhesion. Integrins are the cell adhesion receptors made of a single alpha and a single beta chain with activated and non-activated forms. Integrins such as αvβ_3_ have an important role in tumor growth regulation and angiogenesis as well as in the metastasis. The αvβ_3_ integrins overexpress on a variety of cells, including human malignant melanoma, breast cancer, advanced glioblastoma, and endothelial cells [2, 5].

Nanotechnology is a combining field of science and research that includes chemistry, engineering, biology, and medicine; in recent years, it has become renowned for its strong potential for early diagnosis and targeted cancer treatment. In this regard, a variety of nanoparticles, especially the plasmonic nanostructures, have been nominated as promising theranostic agents [6]. Among the various types of nanoparticle, gold nanostructures with rod morphology have attracted much attention for application in imaging, gene/drug delivery, and hyperthermia therapy due to their characteristic optical and physicochemical properties, high chemical stability, ease of synthesis, and high viability [7–11]. For instance, in the hyperthermia therapy, temperature of the target environment of GNR raises by laser irradiation at a specific wavelength, immediately [12, 13]. Localized heating can affect cancer tissue, providing a noninvasive alternative to conventional treatments such as surgery and radiotherapy [14]. Moreover, GNR has been nominated as a novel nanocarrier system for DNA/RNA delivery due to an ability to provide feasible physical absorption/chemical conjugation of nucleic acids onto its surface [15]. Chakravarthy et al. conjugated ssRNA to GNRs for delivering an innate immune activator against type A influenza virus [16]. Gold nanorods have been employed in immunoassays, and, in the field of biosensing for detection of various biological molecules, too [17–19].

Recently, naked eye detection of human lysozyme has been achieved using heat-induced aggregation of aptamer-functionalized GNR [20]. Due to such widespread and promising medical application of gold nanorods, engineering their physicochemical properties is crucial for determining the effectiveness of GNR in the cell uptake and cytotoxicity [21]. Surface modifications of GNRs include targeting tumors or organs for selective cell binding and entry by receptor-mediated endocytosis [22]. The surface charge of such nanoparticles is also important for its interactions with the cells. Nanoparticles with a positive charge show more uptakes due to interactions between positively charged particles and negatively charged cell membranes [23]. Although applications of GNRs have been fruitful in recent years, the cationic surfactant layer around the nanostructure limits its biocompatibility in the gene/drug delivery strategies [24, 25]. In other types of carrier such as propylene imine dendrimers, the presence of primary amine groups limits nucleic acid transfection that can contribute to toxicity [26].

So far, two of strategies that have been applied for nucleic acid delivery using gold nanoparticles (GNPs) include covalent attachment and electrostatic interaction. The latter one is known to be more attractive than covalent systems, because of using unmodified nucleic acids, including DNA for gene therapy or RNA for knocking down the gene expression.

Researchers have applied some designs for nanoparticle-based vehicles, such as charge-reversal polyelectrolyte-coated GNPs [27], amino acid-functionalized GNPs [28], polyethylenimine (PEI)-functionalized GNPs [29], peptide-functionalized GNPs [30], and cationic lipid-functionalized GNRs [31]. In general, electrostatic-based systems are composed of cationic GNPs that can lead to “proton sponge” effect.

In a recent study, Hirotaka Nakatsuji et al. functionalized GNRs with two conventional lipids, oleate and 1,2-dioleoyl-3-trimethylammonium-propane (DOTAP), and prepared GNR/plasmid DNA complexes to investigate the efficiency of this nanosystem’s transfection. Subsequently, they used near-infrared laser (NIR) illumination for the cells transfected by DOTAP-GNRs for time and site-specific transgene expression [31].

In another interesting approach, Kyung Hyun Min et al. fabricated dipicolyl amine (DPA)-coated GNRs which could make complexes with Zn^2+^ cations. Since Zn^2+^ cation has a great affinity to the phosphate backbone of nucleic acids, Zn(II)/DPA-GNRs could form a stable nanocomplex with siRNA, providing an effective cancer treatment by combination of siRNA and photothermal therapy [32].

In this study, CTAB-GNRs were functionalized by an RGD-HK peptide to increase stability, biocompatibility, and simultaneous targeting of nanostructures. Fourier transform infrared and Raman spectroscopy were utilized to monitor the functionalization process. Toxicity of CTAB-GNRs and peptide-f-GNRs were studied on HeLa cells (as a high integrin) and MDA-MB-231 (as low integrin) cells, using MTT assay. Cellular uptake of GNRs was evaluated by the dark-field microscope and atomic absorption spectroscopy.

## Materials and Methods

### Materials

All chemicals used to synthesize gold nanorods were purchased from Sigma Aldrich (USA). 3-(4, 5-Dimethylthiazol-2-yl)-2,5-diphenyltetrazolium bromide (MTT) was supplied by Roche Applied Science (Indianapolis, IN, USA). Cell culture medium and supplements were supplied by Gibco (Thermo Fisher Scientific).

### Preparation of Gold Nanorods (GNRs)

Gold nanorods (GNRs) were synthesized according to the two-step seed-mediated protocol [33]. Stock solutions of hexadecyltrimethylammonium bromide (CTAB), sodium borohydride, L-ascorbic acid, and silver nitrate were prepared. In the first step, spherical gold nanoparticle seeds were synthesized by an immediate addition of an ice-cold NaBH_4_ solution (600 μl, 0.01 M) to the solution containing CTAB (7.5 mL, 0.1 M) and HAuCl_4_ (250 μl, 0.01 M). Seeds were incubated at 29 °C for 2 h. Growth solution containing 64 μl of 0.01 M AgNO_3_, 9.5 mL of 0.1 M CTAB, and 500 μl of HAuCl_4_ 0.01 M was prepared, and then, 55 μl of 0.1 M fresh ascorbic acid was added. Finally, 30 μl of the seed solution was added to the final solution, gently, and kept overnight. Samples were purified by two rounds of centrifugation at 8000 rpm for 20 min to remove excess cationic surfactant and unreacted gold ions. The precipitate was diluted with deionized water and sonicated to redisperse the GNRs (OD~1.5).

### Estimation of GNR Concentration

Au content was determined using an atomic absorption/flame emission spectrophotometer AA-670. Standard serial dilutions of the gold solution were prepared to obtain the calibration curve. Absorption data and transmission electron microscopy images were considered for determining the concentration of GNRs in this study.

### Functionalization of GNRs

CTAB-GNRs were functionalized by an RGD-HK-peptide sequence to increase stability and biocompatibility and the targeting capability of nanostructures. To this end, we designed a peptide with a cysteine amino acid at N-terminal, a mini PEG inside, a HK repeating stem, and an RGD sequence at the C-terminal. The peptide solutions were prepared at the concentrations of 0.5, 0.75, and 1 mM. Twenty-five microliters of each peptide solution was added to vials of 250 μl GNRs of OD~30. After bath sonication for 30 min, the solution was stirred for 72 h at room temperature, and excess peptides were removed from the functionalized GNRs by centrifugation, twice at 6000 RPM for 20 min.

### Sakaguchi Colorimetric Assay

Based on the presence of an arginine residue in the peptide sequence, Sakaguchi assay was used to determine the average number of peptides attached to each GNR. To make a plot of the standard curve, a series of arginine dilutions were prepared. Then, 10% sodium hydroxide and 1% α-naphthol were added to the sample solution. The absorption intensity was recorded after adding 50 μl of 0.40% urea and 50 μl sodium hypochlorite at 657 nm [34]. The average number of peptides attached to each GNR was estimated by dividing the total amount of bound peptide to the number of GNRs.

### Characterization

The UV–Vis absorption spectra of CTAB-GNRs and peptide-functionalized gold nanorods (f-GNRs) were scanned by a Varian Cary 3E UV–Vis spectrophotometer. FT-IR spectra of CTAB-GNRs and f-GNRs were recorded in the range of 400–4000 cm^−1^_,_ using PerkinElmer 2000 FT-IR spectrometer (USA) with KBr. Raman spectroscopy was monitored in the range of 900–3500 cm^−1^, using Almega Thermo Nicolet dispersive Raman spectrophotometer (USA) at 532 nm of Nd: YLF laser in a liquid nitrogen-cooled environment. Size and morphology evaluations of the gold nanorods were confirmed by Zeiss-EM10C transmission electron microscope (TEM), operated at 80 KV accelerating voltage. Zeta potential of CTAB-GNRs, peptide-f-GNRs, and antisense oligo nucleotides (ASO)-conjugated peptide-f-GNRs was recorded five times for each sample of GNR solution (Zeta sizer 3000, Malvern Instruments, UK).

### Cell Culture

Two different human cancer cell lines of HeLa and MDA-MB-231 were purchased from the National Cell Bank of Iran (Pasteur Institute, Tehran, Iran). Cells were cultured in DMEM medium (Dulbecco’s modified Eagle’s medium, Gibco), supplemented with 2 mM l-glutamine and 10% (*V*/*V*) fetal bovine serum (Gibco), antibiotic solution of 100 U/ml penicillin, and 100 μg/ml streptomycin respectively, maintained at 37 °C in a 5% CO_2_.

### Dark-Field Light-Scattering Images

To study the uptake of functionalized nanoparticles on the HeLa and MDA-MB-231 cells, 3 × 10^4^ of each was seeded on a coverslip and maintained at 37 °C in incubator conditions of 5% CO_2_ for 24 h. The cells were treated with the peptide-f-GNRs (1 nM) or CTAB-GNRs (1 nM) incubated for 2 h. Then, dark-field images were taken under an Olympus microscope (Japan).

### Quantification of the Peptide-f-GNR and CTAB-GNR Complex Uptake

MDA-MB-231 and HeLa cells were seeded onto 24-well plates (10^5^ cells/well). After reaching the confluent of 60–70%, the medium was replaced by CTAB-GNR and peptide-f-GNR suspensions (0.35 nM). After the exposer time of 2 h, all wells were washed with PBS, harvested by a little trypsin incubation, and after addition of 0.2 ml PBS to each well, all contents of each well were transferred to microcentrifuge tubes. The cell suspension was frozen at the temperature of − 20 °C to be used during the analysis. In the following, 0.15 ml of aqua regia (3:1 hydrochloric acid-nitric acid) was added to the cell lysate solution to react for 24 h. Then, the total Au content of these cell suspensions was analyzed by atomic absorption spectroscopy (AAS); (the experiments were conducted three times to be sure of reproducibility from data and reported as an error bar). According to the known dimension of the GNRs through TEM, the number of nanoparticles per well was calculated. For calculation of the uptake percentage of GNRs in the cells, the number of GNRs within the cells divided into the total amount of GNRs was applied to the cells.

### Cytotoxicity Assay

Cytotoxicity of the peptide-functionalized GNRs compared to CTAB-GNRs on cancerous cell lines was evaluated using the MTT assay. The HeLa and MDA-MB-231 cells were treated with the GNR suspension functionalized by different concentrations of peptide solutions (0.5, 0.75, and 1 mM) and CTAB-GNRs in concentrations of 1 nM, 5 nM, 25 nM, and 125 nM. The experiments were conducted at least three times to be sure of reproducibility from data and reported as average ± standard deviation; in case of histogram, the standard deviation was reported as an error bar. The best functionalization model was obtained by crossover design to determine the best concentration of GNR.

### Statistical Analysis

Data were analyzed using the general univariate linear model followed by Tukey and Duncan’s multiple comparison tests to compare all groups in terms of statistical difference. A *P* value of < 0.05 was regarded as statistically significant. The Statistical Package for the Social Sciences package (SPSS, Chicago, IL, USA, version of 16.0) was used for data analysis.

## Results

### CTAB-GNRs and Peptide-f-GNR Characterizations

#### UV–Vis Spectroscopy

Due to the anisotropic shape of GNRs, they depict two surface plasmon resonance (SPR) bands in the visible and near-infrared regions, being characteristic of the oscillation of the conduction band electrons of the nanorods in width (transverse SPR) and length (longitudinal LSPR), respectively. Intensity and position of the latter (LSPR) are mainly dependent on the yield and aspect ratio of the nanostructures [35].

**Fig. 1:**
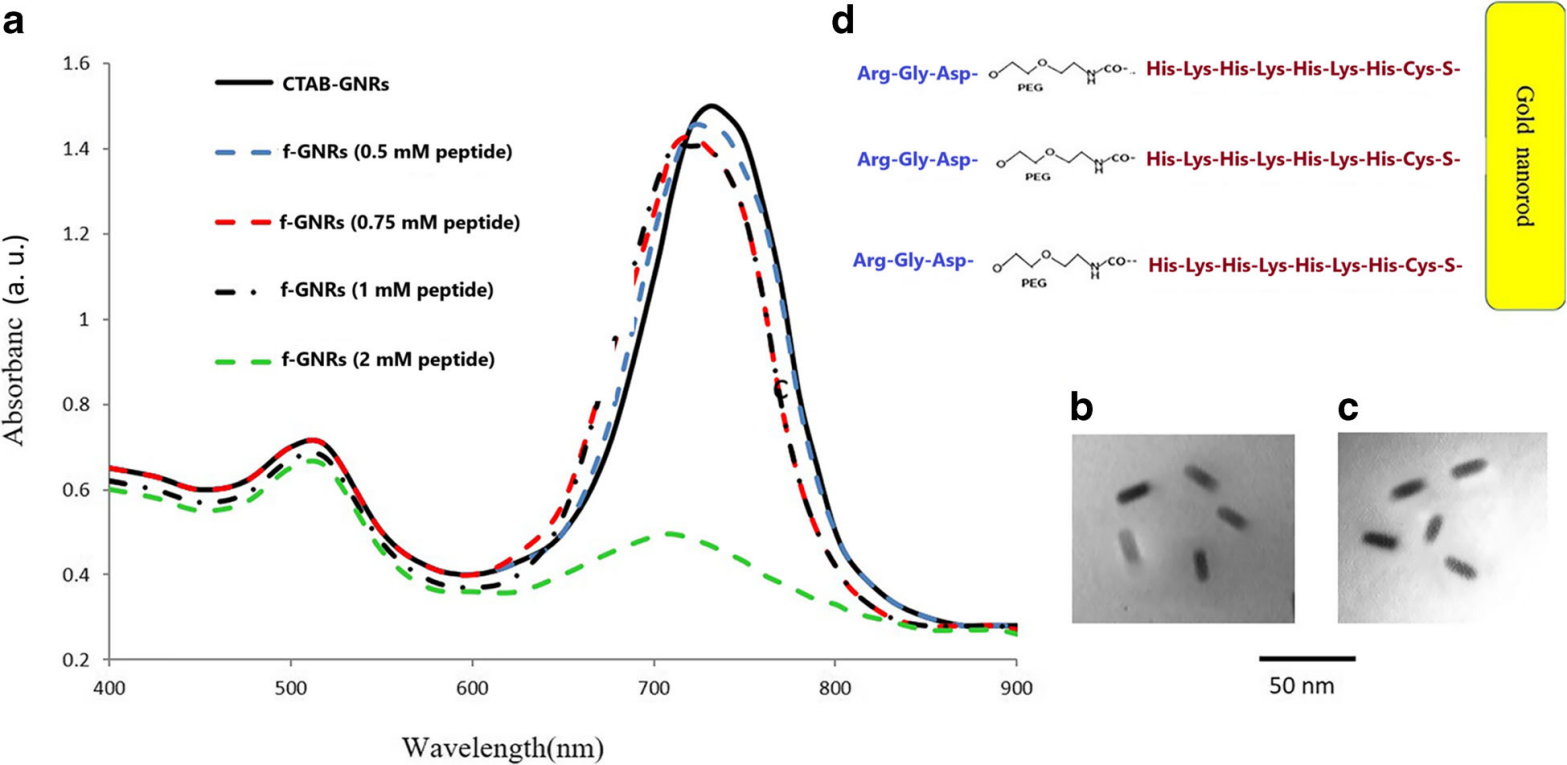
**a** UV–Vis absorption spectra of CTAB-GNRs (black solid line) and peptide-functionalized GNRs using different peptide concentrations (dashed lines). TEM image of CTAB-GNRs (**b**) and peptide-f-GNRs (**c**), Scale bar: 50 nm. Schematic diagram of peptide-conjugated GNRs (**d**)

The characteristic SPR bands of CTAB-GNRs and peptide-functionalized GNRs (using 0.5, 0.75, 1, and 2 mM peptide) appeared at 520 and 720 nm (Fig. 1a). A decrease of LSPR intensity with a shift in the position of plasmonic band depicted interaction of the nanostructures with the biomolecules. Transmission electron microscopy confirmed the rod morphology of the nanostructures, giving the aspect ratio of the GNRs to be 3.3 (20 × 6), while the size and shape of the nanoparticles have not changed dramatically after functionalization with the desired peptide (Fig. 1b, c). As shown in Fig. 1a, the peptide concentration of 2 mM caused a sharp drop in LSPR absorption peak due to nanorod deformation. Therefore, for the other tests, this concentration eliminated. Figure 1d shows a schematic image of the nanostructure (peptide-f-GNR).

#### Zeta Potential Analysis

**Fig. 2:**
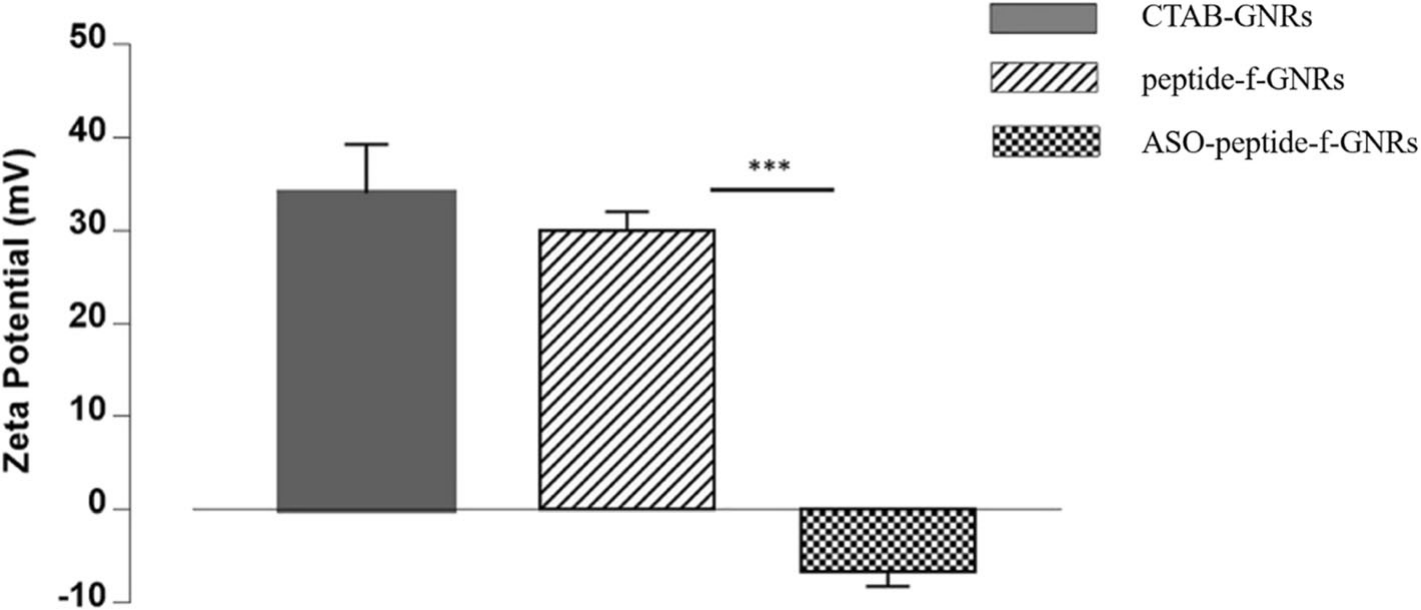
Zeta potential of CTAB-GNRs (+ 35 mV), peptide-functionalized GNRs (+ 29 mV), and antisense oligonucleotides (ASO-conjugated GNRs (− 6 mV). The results represent the mean and standard deviation (sd) of three independent experiments (***P < 0.001)

Measurements of the zeta potential of the CTAB-GNRs and peptide-f-GNRs were taken using a laser Doppler electrophoresis. Results showed that the zeta potential of the peptide-f-GNRs reduced slightly compared to CTAB-GNRs (Fig. 2). Changes in the value of zeta potential can give an idea about the surface charge of the nanostructures before and after any functionalization or surface modification processes. In this investigation, in spite of the removal of CTAB after several rounds of centrifugation, the zeta potential analysis of GNRs still shows positive value (+ 35 mV) due to the presence of the cationic surfactant around the nanostructures. Upon the surface modification and ligand exchange, the zeta potential value of peptide-functionalized GNRs decreased to 29 mV which shifted to negative value after incubation with antisense oligonucleotides (− 6 mV), confirming bioconjugation of peptide-f-GNRs to ASO (as shown in Fig. 2). Therefore, zeta potential analysis can be considered as a useful strategy to monitor the changes in the surface charge/environment of the nanostructures after functionalization with biomolecules of interest.

#### Fourier Transform Infrared Spectroscopy

**Fig. 3:**
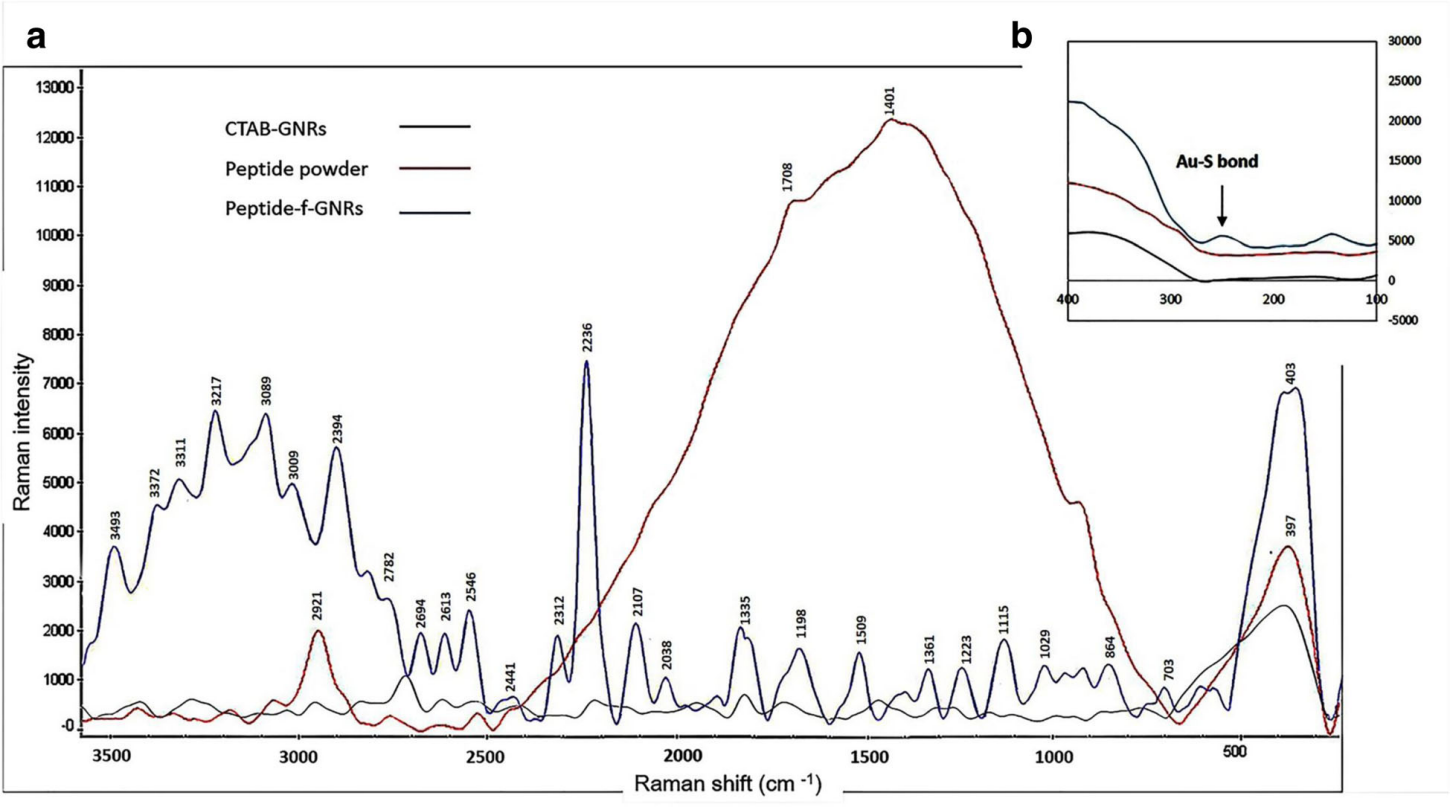
FTIR spectra of peptide-f-GNRs compared to CTAB-GNRs and peptide powder

Infrared spectroscopy relates to the molecular vibrations of specific functional groups. The energy of the most active molecular vibrations (twisting, stretching, and rotating) correlated with electromagnetic spectrum in the infrared region. Many vibrational modes do not show a single type of bond oscillation but are strongly reliant on adjacent bonds and functional groups. The FTIR spectra of GNRs before and after functionalization of the nanostructures are shown in the Fig. 3. Bands in the 1675–1680 cm^−1^ region were related to the presence of carbonyl vibration (C=O). These results were in good agreement with IR spectra of a typical amino acid. Comparison between FTIR spectra of the peptide powder and peptide-f-GNRs shows that the functionalization reaction was successful (Fig. 3). The characteristic band of the SH group in peptide powder spectrum (2550 cm^−1^) disappeared in peptide-f-GNRs, owing to its conjugation to Au [36]. Furthermore, an appearance of severe characteristic bands of PEG at 2830 cm^−1^ (C–H stretching mode) and 1150 cm^−1^ (C–O–C stretching modes) [37] in the peptide-f-GNR spectrum (also seen in the peptide powder spectrum) confirmed the bioconjugation.

#### Raman Spectroscopy

**Fig. 4:**
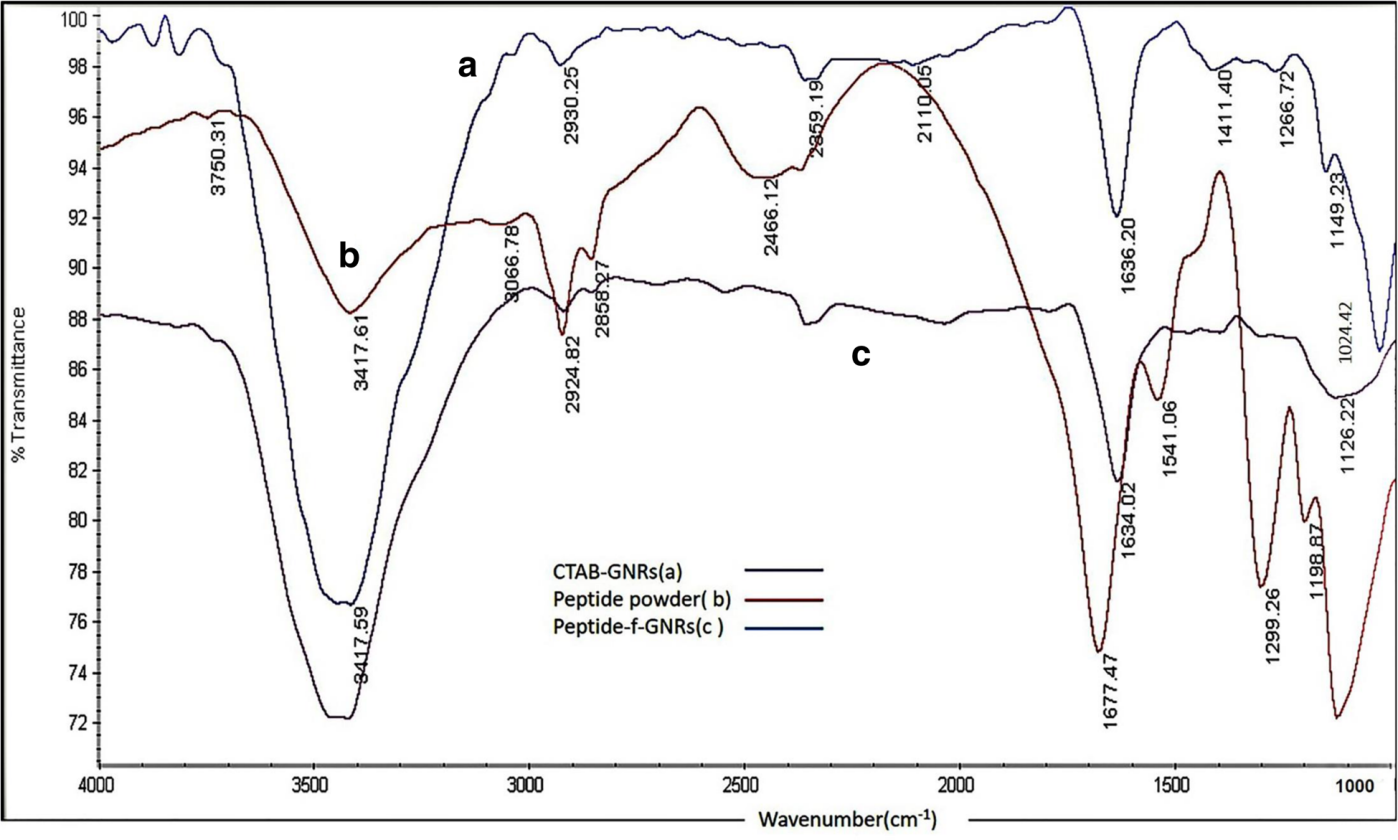
SERS of peptide on the gold nanorods and Raman spectrum of peptide powder and CTAB-GNRs at 250–3600 cm^−1^ region (**a**) and 100–500 cm^−1^ region (**b**)

One of the properties of noble metal nanoparticles that has attracted significant research attention is their ability to act as a surface-enhanced Raman scattering (SERS) substrate. SERS allows 10^2^–10^14^-fold or more enhanced detection of weak Raman signals of molecules near a specific type of surface. According to the previous study, SERS spectra of the peptide adsorbed molecules may not look exactly like the original Raman spectrum, because only the residues near the Au surface will be enhanced [38]. In this study, Raman spectroscopy was exploited to monitor different modes of vibration in peptide and peptide-f-GNRs. Comparison of the peptide Raman spectrum and the SERS peptide-f-GNR spectrum indicated (Fig. 4) that intensity of many prime vibration bands has been amplified extraordinarily by the SERS-active GNRs. In contrast, only a few weak Raman peaks could be observed in the peptide powder without the Au colloids due to the fluorescence background coating Raman signals. The fluorescence background can be repelled clearly in the SERS spectra, especially in the 700–2500 cm^−1^ region, and we can observe significant Raman bands of peptide-f-GNRs, evidently. Compared with the peptide powder sample, the peptide-f-GNRs exhibited remarkable SERS signals at 350 cm^−1^, 373 cm^−1^, 1819 cm^−1^, 2079 cm^−1^, 2134 cm^−1^, 2187 cm^−1^, 2261 cm^−1^, 2305 cm^−1^, 2375 cm^−1^, 2474 cm^−1^, 2532 cm^−1^, 2644 cm^−1^, 2724 cm^−1^, 2773 cm^−1^, and 2869 cm^−1^.

In particular, the peptide-f-GNR sample represented SERS peaks of higher intensity in 2100–2900 cm^−1^ region. Figure 4b also compares the CTAB-GNRs and peptide-f-GNRs showing an appearance of a new peak at 260 cm^−1^ in the peptide-f-GNR spectrum. This might be correlated to the Au–S bond according to a recent study. The amide III C–N stretching at 1259 cm^−1^ is not clear in the peptide powder but seen in peptide-GNRs. The histidine vibrations at 878, 1490, and 1500 cm^−1^ are also observed in the peptide-bound onto GNRs. The signal at 831 cm^−1^ is assigned to the lysine C–C stretch, and the 1045 cm^−1^ signal is assigned to C–N stretch vibrations from lysine [39]. The CTAB-coated GNRs show strong Raman band at 380 cm^−1^, which could be related to the Au–Br vibration, due to the closer distance to GNRs’ surface. According to Fig. 3, this band is still observed in the Raman spectrum of the peptide-f-GNRs due to the existence of some CTAB molecules. However, the intensity of this band in the peptide-f-GNRs is much weaker due to the replacement of some CTAB molecules with a thiolated RGD-HK peptide.

#### Sakaguchi Assay

Sakaguchi assay was applied to estimate the number of the peptide molecules conjugated to each GNR. Alpha-naphthol is a Sakaguchi agent that reacts specifically with guanidine residue on the side chain of arginine. Thus, α-naphthol can be used to determine the amount of arginine-containing peptides on the peptide-f-GNRs. In this test, we used a dilution series of arginine amino acid solutions to prepare the standard curve. Since the peptide only had one arginine molecule, calculation of the number of the peptides bound to each GNR was possible using this test. In fact, each peptide molecule could be considered as one arginine. Therefore, based on the protocol, 25 μl of the peptide solutions (0.5, 0.75, and 1 mM) were incubated with 250 μl CTAB-GNRs (OD~30). Considering the average concentration of nanorods to be 30 nM in OD~1 (according to ICP and TEM images), the final concentration of GNRs for this assay is 930 nM.

Taking the concentration of both nanostructures and peptide molecules (0.75 mM) into account, there would be 95 peptide molecules in the solution for each nanorod. To estimate the number of peptide molecules attached to each GNR, samples were centrifuged at 6000 RPM for 20 min after the incubation.

The precipitation consisted of the peptide-functionalized GNRs, and the unbound peptides were present in the supernatant. Based on the standard curve, concentration of the peptide in the supernatant was calculated. It was estimated that approximately 54 peptide molecules were attached to each GNR, by subtracting the number of unbound peptides in the supernatant from the amount in the initial solution. Thus, the percentage of peptides bound to each GNRs was calculated to be 57%.

To monitor the possible effect of peptide concentration on its binding affinity to GNRs, this test was also tried with other concentrations of peptide solutions, i.e., 1 and 0.5 mM. It was noted that relatively similar number of peptides (50–60) were bound to each GNR. Therefore, it can be concluded that the nanostructures in this study have a specific capacity to bind to peptide molecules, and within this range of peptide concentration, there are not any remarkable changes in the number of peptides available on the matrix of GNRs. Further experiments in this investigation were carried out with 0.75 mM peptide solution for the functionalization process.

### Biological Studies

#### Light-Scattering Images of the Peptide-f-GNRs Inside the Cells

**Fig. 5:**
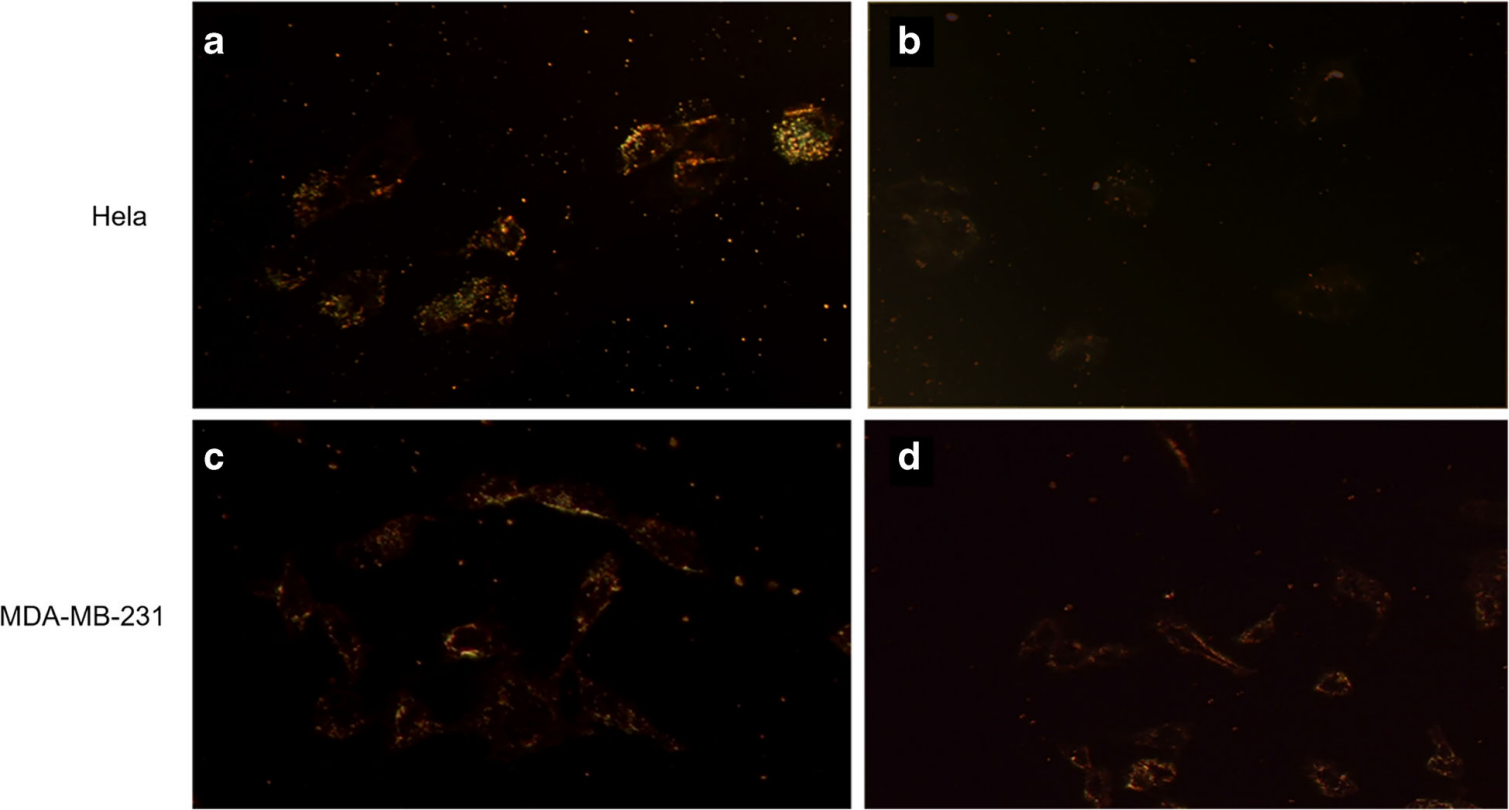
Dark-field light-scattering images of two cell lines at × 20 magnification after incubation with CTAB-GNRs and peptide-f-GNRs for 2 h. The HeLa (as cancer cells with high level of αvβ3 integrins) cells incubated with peptide-f-GNRs (**a**) and CTAB-GNRs (**b**). The MDA-MB-231 (as cancer cells with low level of avb3 integrins) cells incubated with peptide-f-GNRs (**c**) and CTAB-GNRs (**d**). It seems that peptide replacement promotes the cellular uptake of peptide-f-GNRs

The potential of nanostructures to permeate into specific cells was examined by the dark-field light-scattering technique of imaging. Figure 5 shows dark-field images of the HeLa (as high integrin) and MDA-MB-231(as low integrin (cells after incubation with peptide-f-GNRs and CTAB-GNRs for 2 h. As shown in the Fig. 5, proper internalization of peptide-f-GNRs within the Hella cells was observed in dark-field images. Previous reports have shown that the cationic ligands, such as oligopeptides, attain high affinity with the plasma membrane, allowing better cellular uptake through endocytosis, too [23, 40, 41].

#### Intracellular Uptake of GNP Measured with AAS

Atomic absorption spectroscopy (AAS) is a more accurate technique for quantification of internalized GNRs to all exposed cells, while dark-field images only show individual cells [42]. In addition, sensitivity of this method is higher than imaging due to the ability of detection of internalized GNRs in ppb level [43].

Therefore, we analyzed the percentage uptake of CTAB-GNRs and peptide-f-GNRs into the HeLa and MDA-MB-231 cells as a function of total GNRs administrated.

**Fig. 6:**
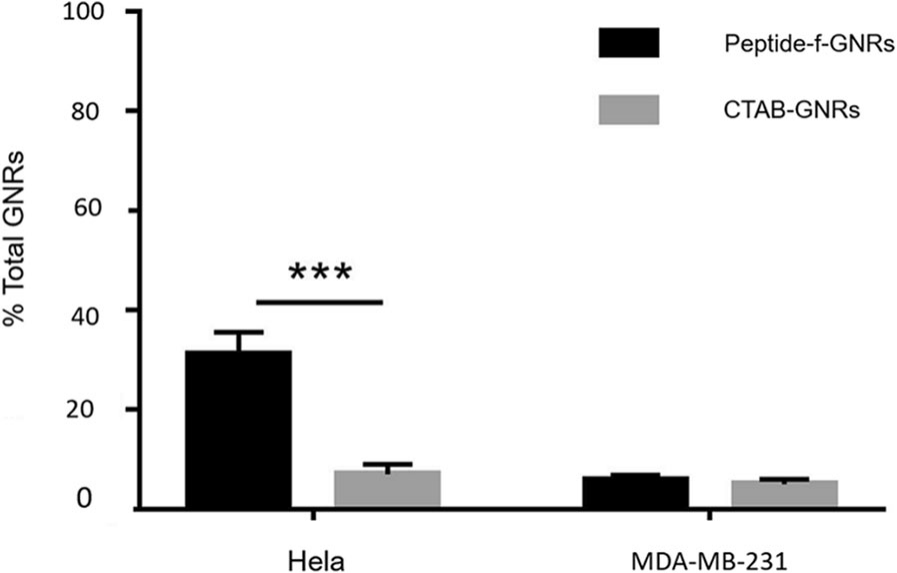
Quantification of internalized GNRs (CTAB-GNRs and peptide-f-GNRs) in HeLa and MDA-MB-231 cells by atomic absorption spectroscopy (AAS). The cells were incubated with 0.35 nM GNRs for 2 h. After washing the cells extensively, and lysing by aqua regia, the total gold content in the different wells was measured by AAS. HeLa and MDA-MB-231 cells showed different uptake behavior.*P < 0.05; **P < 0.01; ***P < 0.001

As demonstrated in Fig. 6, a large number of peptide-f-GNRs internalized into the HeLa cells compared with the MDA-MB-231 cells. Furthermore, the HeLa and MDA-MB-231 cells internalize less of CTAB-GNRs than the initial value administrated (< 5%). Based on the dark-field imaging, Fig. 6 shows higher internalization of peptide-f-GNRs into HeLa cells compared with CTAB-GNRs, while images shown in Fig. 5 illustrate that the shining of CTAB-GNRs in both cell lines was the same approximately. These results were confirmed by AAS data due to determining of the percent internalization of CTAB-GNRs and peptide-f-GNRs. After exposure time of 2 h to GNRs, 28.4 and 11.1% of the total number of the applied peptide-f-GNRs were uptake in HeLa and MDA-MB-231, respectively.

#### Cytotoxicity Assay

**Fig. 7:**
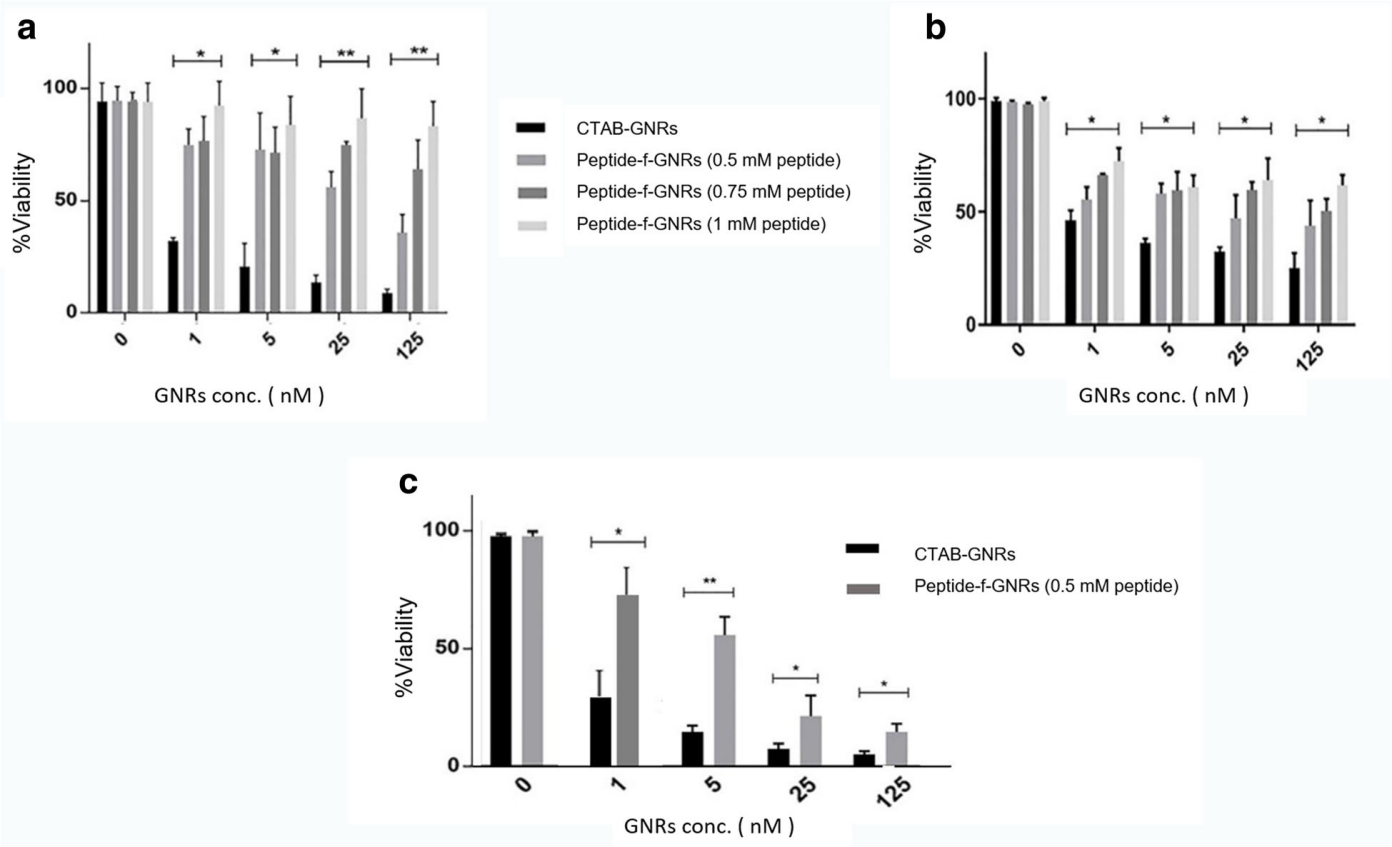
Cell viability (MTT) assay after incubation of HeLa (**a**) and MDA-MB-231 (**b**) cells with peptide-f-GNRs (using different concentrations of peptide) compared with CTAB-GNRs after 24 h. Cell viability after incubation of HeLa cells with peptide-f-GNRs compared with CTAB-GNRs after 48 h (**c**). The results are expressed as percentages compared with untreated control cells and represent the mean and standard deviation (sd) of three independent experiments (***P < 0.001)

MTT assay was applied to assess the toxic effect of nanostructures on cells. To obtain the optimum concentration of peptide to increase biocompatibility of the nanocarrier, a serial dilution of peptide solutions (0.5, 0.75, and 1 mM) were tested for functionalization of the nanorods. GNR at nanorod concentrations of 1 to 125 nM was incubated with the HeLa and MDA-MB-231 cell lines. All experiments were conducted in triplicate. As shown in Fig. 7a, b), at lower concentrations of GNRs, there is a less difference between the cytotoxic effect of the functional and pristine/nonfunctional GNRs (the cytotoxic effect of the peptide-functionalized GNRs is about half of the nonfunctional GNRs), which can be due to the toxicity of the cationic surfactant (CTAB) molecules in pristine GNRs.

**Fig. 8:**
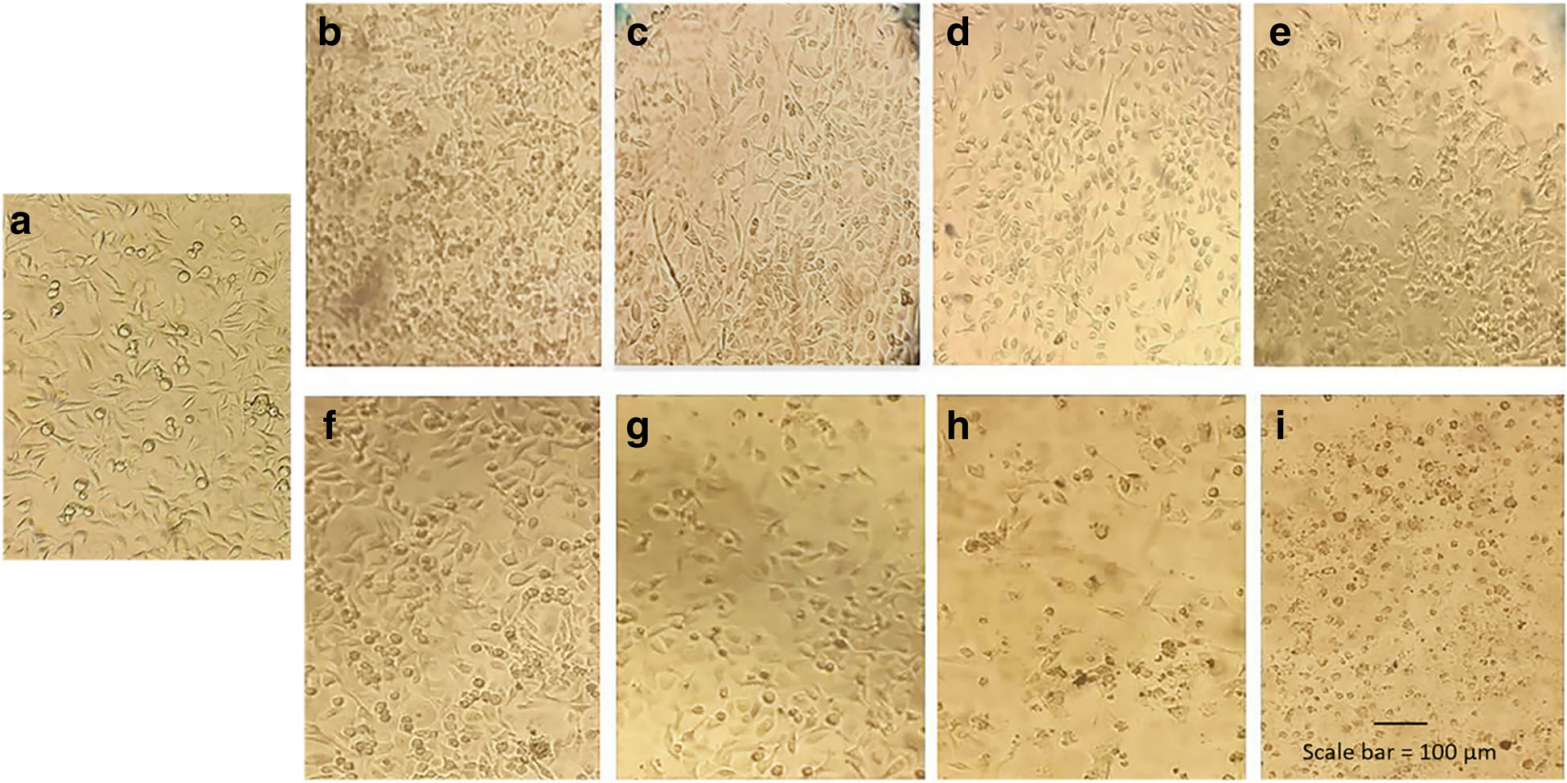
Light microscope image (× 10) of cells treated with GNRs. HeLa-untreated cells (**a**), HeLa treated by 1 nM of CTAB-GNRs (**b**), 5 nM of CTAB-GNARs (**c**), 25 nM of CTAB-GNARs (**d**) and 125 nM of CTAB-GNARs (**e**) with less viability, and 1 nM of peptide-f-GNRs (**f**), 5 nM of peptide-f-GNRs (**g**), 25 nM of peptide-f-GNRs (**h**), and 125 nM of peptide-f-GNRs (**i**) with more viability. The cell density decreased, and the morphology changed in CTAB-GNR-treated cells in comparison with cells treated with peptide-f-GNRs

However, at higher concentrations (up to 125 nM), the difference between the cytotoxic effects of the functional and nonfunctional GNRs is much higher. In fact, at higher concentrations, functional GNRs could remarkably reduce the cytotoxicity of GNRs. The cytotoxic effect of the peptide-f-GNRs on Hella cell line decreased up to six times compared to that of CTAB-GNRs. Of course, the MDA-MB-231 cells showed different results. In fact, in this cell line, with 125-nM peptide concentration, the cytotoxic effect of the functional GNRs is still less than that of CTAB-GNRs. Nevertheless, there is less difference between the cytotoxic effects of the samples. This can be due to the fact that toxic response of different cell lines is different toward these nanostructures. Cell density and morphology were also checked by microscopic images which could confirm cytotoxicity results. The images demonstrated the effect of the peptide-f-GNRs and CTAB-GNRs on the cell morphology and viability (Fig. 8(. The peptide-f-GNRs showed less cytotoxic effect on density and morphology of the cells compared to CTAB-GNRs of equal concentration.

## Discussion

Gold nanorods (GNRs) have been nominated as a promising candidate in theragnostic applications, such as combining imaging, gene/drug delivery, and photothermal therapy. GNRs are commonly synthesized in the presence of a cationic surfactant (CTAB) that limits its biocompatibility and biomedical utility. Several surface engineering strategies have been applied to date, proposed to reduce the toxic effect of GNRs, such as coating, layer-by-layer, and ligand exchange. Such methods, however, can have affect cell uptake in other ways by promoting or hindering the internalization process [44].

On the other hand, the use of RGD sequence for targeting of nanoparticles to the invasive tumors has been investigated, extensively [45, 46]. However, in the previous studies, two forms of linear or cyclic of this ligand was attached to the surface of nanoparticles through PEG molecule, and usually, functionalization and targeting have been done using separated molecules [47, 48].

In this research, we designed a novel peptide to exchange the CTAB ligands around the GNRs. The peptide was conjugated to GNRs through Au–S bonds between the cysteine residue of the peptide and the surface of GNRs. There are many reports of biomolecules conjugation to gold nanoparticles through Au–S bonds [36, 49].

The cysteine-terminated peptide (C(HK)_4_-mini PEG-RGD) and CTAB-GNR solution was incubated for up to 72 h. The accuracy of peptide binding to nanoparticles was confirmed by UV–Visible, Raman, and Ft-IR spectroscopy techniques and measuring zeta potential. As shown in Fig. 1, there is a small blue shift in the longitudinal surface plasmon resonance band (LSPR) after replacement of excess CTAB molecules, with negligible changes upon the increment of peptide concentrations of 0.5, 0.75, 1, and 2 mM. The reduction in the intensity of LSPR band represents the interaction between the surface of nanostructures and the peptides, without disruption of rod morphology; except for a decline in the second peak by using 2-mM peptide concentration. Subsequently, the toxicity of the nanoparticles was analyzed using MTT assay.

It is worth mentioning that nanoparticle toxicity depends on a number of important factors, such as the type, size, shape, aspect ratio, surface charge, hydrophobicity, and composition of material [23, 50]. The cytotoxic property of nanoparticles could be due to the presence of toxic compounds, solubility, or contamination [51]. Biological factors such as the type of treated cell and exposure conditions (nanoparticle concentration, medium, composition, cell confluence, and temperature) can also affect cytotoxicity [28, 29]. Although it has generally been proven that positive charged nanoparticles can damage the membrane of the cell more than their negative charged counterparts, the relationship between surface load and toxicity is a complex process and depend on a set of factors such as, composition, size, shape, and density of the surface charge of nanoparticles [52]. Nanoparticles can enter the cells through passive or active transport. One of the ways that can prevent cell membrane damage is to target nanoparticles to enter the cell through endocytosis mechanisms [53, 54].

After incubation of both cell lines with peptide-f-GNRs and CTAB-GNRs, it was revealed that the RGD-mini(PEG)-(HK)_4_-peptide presents a useful alternative for functionalization of GNRs as it reduces the cytotoxic effect of the cationic surfactant in both cell lines. However, no significant variation was observed for different concentrations of the peptide in the cytotoxicity reduction of peptide-f-GNRs for MDA-MB-231 cells; increasing the peptide concentration reduced cytotoxicity of the constructed nanostructure in the HeLa cells. This may be related to the existence of more integrin receptors on the cell membrane of the HeLa cell line.

In addition, inverted microscopic images revealed the effect of CTAB-GNRs and peptide-f-GNRs on cell morphology and viability. In comparison with CTAB-GNRs, peptide-f-GNRs of the same concentration showed less cytotoxic effect on cell density and morphology. Based on dark-field images and atomic absorption spectroscopy of two cell lines incubated with CTAB-GNRs and peptide-f-GNRs, it seems that the latter one has been internalized into the HeLa cells more than that of CTAB-GNRs through receptor-mediated endocytosis, being mainly distributed in the cytoplasm. In addition, entry of the nanoparticles into the MDA-MB-231 cells was also evident. This phenomenon could be due to the enhanced permeability and retention (EPR) effect [55]. The ability of peptide-f-GNRs to conjugate to antisense oligonucleotides (ASO) was also confirmed using zeta potential, which was due to the repeated HK (His-Lys) sequence inside the peptide. The ability of HK polymers as noncovalent nucleic acid carriers has already been proven. In histidine-lysine polymers, lysine causes electrostatic interaction with negatively charged nucleic acid phosphates, and histidine, with its buffering effect, plays an important role in nucleic acid endosomal escape [56, 57].

## Conclusion

We successfully developed a peptide-functionalized GNR for proper biocompatibility, targeting, and nucleic acid delivery, simultaneously. Overall, it seems that the RGD peptide promoted cellular uptake of the GNRs. Finally, in only one-step ligand exchange, we were able to achieve a less toxic and targeted biocompatible nanoparticle with the ability of electrostatic interaction to nucleic acids. We hope this nanostructure could further provide applicability of the nanocarriers in a variety of biological applications, such as, negatively charged anticancer gene/drug delivery, i.e., diagnosis and targeted therapy of cancerous tumors.

## Abbreviations

C(HK)4-mini PEG-RGD: Cys(His-Lys)_4_-mini(Polyethylene Glycol)-Arg-Gly-Asp
CTAB: Hexadecyltrimethylammonium bromide
FT-IR: Fourier transform infrared
GNPs: Gold nanoparticles
GNRs: Gold nanorods
HK: Histidine-lysine
MTT: 3-(4,5-Dimethylthiazol-2-yl)-2,5-diphenyltetrazolium bromide
OD: Optical density
PEG: Polyethylene glycol
Peptide-f-GNRs: Peptide-functionalized gold nanorods
RGD: Arg-Gly-Asp
SPSS: Statistical Package for the Social Sciences
TEM: Transmission electron microscopy
